# Modulating Intrinsic Connectivity: Adjacent Subregions within Supplementary Motor Cortex, Dorsolateral Prefrontal Cortex, and Parietal Cortex Connect to Separate Functional Networks during Task and Also Connect during Rest

**DOI:** 10.1371/journal.pone.0090672

**Published:** 2014-03-17

**Authors:** Jennifer K. Roth, Marcia K. Johnson, Fuyuze Tokoglu, Isabella Murphy, R. Todd Constable

**Affiliations:** 1 Department of Social Sciences, Concordia College New York, Bronxville, New York, United States of America; 2 Department of Psychology, Yale University School of Medicine, New Haven, Connecticut, United States of America; 3 Magnetic Resonance Research Center, Department of Diagnostic Radiology, Yale School of Medicine, New Haven, Connecticut, United States of America; 4 Child Study Center, Yale University School of Medicine, New Haven, Connecticut, United States of America; 5 Departments of Biomedical Engineering, and Neurosurgery, Yale University School of Medicine, New Haven, Connecticut, United States of America; Penn State University, United States of America

## Abstract

Supplementary motor area (SMA), the inferior frontal junction (IFJ), superior frontal junction (SFJ) and parietal cortex are active in many cognitive tasks. In a previous study, we found that subregions of each of these major areas were differentially active in component processes of executive function during working memory tasks. In the present study, each of these subregions was used as a seed in a whole brain functional connectivity analysis of working memory and resting state data. These regions show functional connectivity to different networks, thus supporting the parcellation of these major regions into functional subregions. Many regions showing significant connectivity during the working memory residual data (with task events regressed from the data) were also significantly connected during rest suggesting that these network connections to subregions within major regions of cortex are intrinsic. For some of these connections, task demands modulate activity in these intrinsic networks. Approximately half of the connections significant during task were significant during rest, indicating that some of the connections are intrinsic while others are recruited only in the service of the task. Furthermore, the network connections to traditional ‘task positive’ and ‘task negative’ (a.k.a ‘default mode’) regions shift from positive connectivity to negative connectivity depending on task demands. These findings demonstrate that such task-identified subregions are part of distinct networks, and that these networks have different patterns of connectivity for task as they do during rest, engaging connections both to task positive and task negative regions. These results have implications for understanding the parcellation of commonly active regions into more specific functional networks.

## Introduction

Areas within supplementary motor area (SMA), the inferior frontal junction (the junction of the inferior frontal sulcus and precentral sulcus; IFJ), superior frontal junction (the junction of the superior frontal sulcus and precentral sulcus; SFJ) and parietal cortex are active in a wide variety of cognitive tasks [2], [3], and are part of what has been termed the “task positive network” [4]. Activations of SMA, IFJ, SFJ, and parietal cortex are so common during cognitive tasks that it suggests either that these regions serve a similar general function regardless of task, or that there are subregions, not yet identified, within these regions that are functionally dissociable.

Early reviews of executive function [5], [6] did not differentiate subregions within each of these areas. However, more recently, immediately adjacent functional subdivisions of SMA, IFJ, SFJ and parietal cortex were found to respond to different aspects of executive function, for example, different aspects of perceptual attention [7], [8], [9], refreshing vs. updating working memory [1], [10], perceptual attention switching versus switching attention in working memory [11], [12], old versus new memory judgments [13], and switching categorization rules [14].

Functional and structural connectivity results also support the notion that large, commonly active regions contain subdivisions involved in different networks and tasks. Gilbert, Henson and Simons (2010) [15] showed functional subdivisions of anterior medial PFC just millimeters apart across cognitive tasks. Johansen-Berg et al. (2004) [16] found that the pre-SMA had structural connections to cognitive regions (inferior frontal, medial parietal), whereas SMA connected to precentral gyrus, motor and premotor regions.

Even at rest, connectivity patterns change when the seed voxel is moved a small distance. Cohen, Fair, Dosenbach et al. (2008) [17] found a dramatic and abrupt change in functional connectivity in rest data when a seed was moved a small distance in angular or supramarginal gyrus. Thus, connectivity analysis is a promising way to further explore potential networks associated with directly adjacent regions of cortex.

A number of studies have found that network connectivity observed in tasks is also detectable in resting state [18], [19], [20], [21], [22], [23], [24], [4], [25], [26], sleep [27], [28] and under anesthesia where consciousness is minimized [29], [30], [31], [32], [33].In addition, there is evidence that functional connections at rest are modulated by engagement in tasks [21].Thus resting state networks are sometimes called *intrinsic* networks.

As yet unexplored is the degree to which task-identified subregions are part of distinct networks. Also, it is not known if these networks are intrinsic, that is, are manifest in significant resting state connectivity. Furthermore, what is the nature of the network reconfiguration when participants engage in a specific task?

This paper addresses how the functional connectivity seen for such more specific subregions of areas commonly found in working memory tasks relates to the functional connectivity seen for these subregions at rest. That is, are the same or different whole-brain connectivity patterns identified in task and rest for subregions of SMA, IFJ, SFJ, and parietal cortex? Do these network connections represent intrinsic connections or connections that form only in the service of the task? Subregions were identified in a previous study that compared brain activity associated with two executive functions engaged in working memory tasks, *updating* and *refreshing* [1]. Shen, Papademetris and Constable (2010) [34] used a data-driven machine learning segmentation approach on the intraparietal sulcus data from Roth et al. to identify clustered voxels based on the similar temporal patterns and found similar functional subunits, further validating the parcellation of cortex reported by Roth et al. In the present study, we used the functional subdivisions of major regions (SMA, IFJ, SFJ and left and right parietal cortexes) as seeds in a series of functional connectivity analyses. We regressed out the mean event related task responses, then collapsed across updating and refreshing task blocks to select seeds associated with working memory function. This procedure reduces any bias that might occur from selecting seeds based on their event related responses to task-specific events then calculating connectivity based on those same data. We then assessed whether there was similar significant functional connectivity at rest in these networks in an independent set of participants.

## Methods

To test the degree to which task-identified subregions are part of distinct networks, a series of functional connectivity analyses were performed to confirm that these task-related subdivisions of SMA, IFJ, SFJ, and parietal cortexes are connected to different functional networks. Furthermore we tested if these networks are intrinsic, by exploring whether they are also connected during the resting state. We also explored the nature of the network reconfiguration when participants engaged in task versus rest.

### Ethics statement

Participants were compensated and all gave written informed consent. The experiments were undertaken with the approval of Yale University School of Medicine Human Investigation Committee, and in compliance with national legislation and the Code of the World Medical Association for Ethical Principles for Medical Research Involving Human Subjects.

### Participants

Participants in the update and refresh conditions were 22 (12 females) healthy adult (mean age = 24 yrs, range 19–44) non-smokers, with no history of head injury, psychiatric illness, drug or alcohol abuse, and no current medications that would affect the function of the brain, heart or blood circulation. Participants in the resting state task were 45 right handed healthy adults (21 females, mean age 32.2 years, range 20–45). Participants were compensated and all gave written informed consent. The experiments were undertaken with the approval of Yale University School of Medicine Human Investigation Committee, and in compliance with national legislation and the Code of the World Medical Association for Ethical Principles for Medical Research Involving Human Subjects. Data using the update and refresh tasks were previously reported in Roth et al, 2009, which showed parcellation of major regions of the brain into subregions during working memory. The resting state data were reported previously in a separate analysis [34].

### Procedure for update and refresh scan session

The procedures are described in detail in Roth et al (2009) [1]. Briefly, there were 6 fMRI experimental runs, each with 4 blocks each of update and refresh tasks, presented pseudorandomly and counterbalanced within run and across scan session. Update and refresh blocks were nearly identical in visual input and timing. Words appeared on the screen one at a time and participants were instructed to read them silently.

#### Update blocks

**Figure 1:**
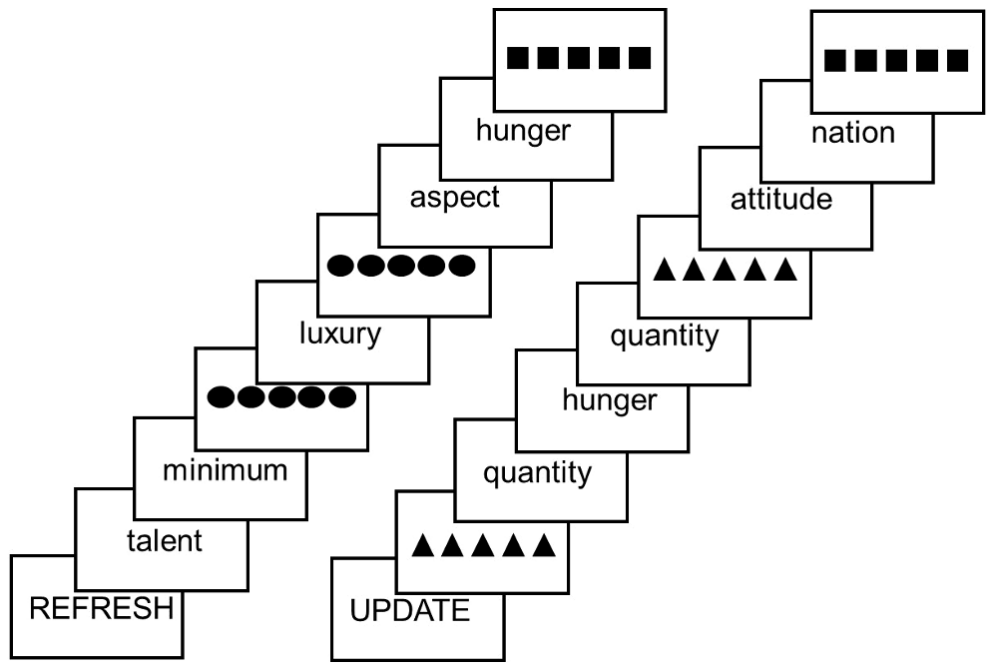
Update and refresh tasks for the task scanner runs. Participants saw words appear on the screen one at a time. When participants engaged in the Refresh task they were asked to read each word. When they saw the row of dots (refresh cue) they were instructed to think back to the last word they read (refresh) only once, then continue to read the subsequent word. When participants engaged in the Update task they were asked to remember one word at a time in working memory. If they saw the word they were remembering they were to press a button. When they saw a row of triangles (update cue) they were to forget the word they were maintaining in working memory and remember the word they saw after the triangles. Again, if they saw this word subsequently in the stream of words they were to press a button indicating it matched the contents of working memory.

Participants saw a word and were cued to maintain it in WM and sometimes were cued (with a row of triangles) to replace the word currently being held in WM with the next word to appear on the screen (*update*; see Figure 1). Thus, in update blocks, participants always maintained one word in WM while reading other words, and their task was to determine whether each word they read matched or did not match the word being maintained. If the word on the screen matched the word in WM, participants responded with a button press (*match*); otherwise, they did not press a button. Occasionally they saw a row of squares, acting as a sensory control event (*control*), and they were instructed to look at the squares, but continue to maintain the current word in WM and wait for the next word.

#### Refresh blocks

As in update blocks, single words appeared on the screen one at a time and participants were instructed to read them. Occasionally, instead of a word, they saw a row of dots which cued them to think back to the word that preceded the refresh cue (*refresh* event; see Figure 1). They were instructed to think back to that word once, and not continue to think of it. Occasionally they saw a row of squares that cued them to look at the squares and wait for the next word; this served as a sensory *control* event in which they saw a row of geometric stimuli, as on Refresh trials, but were not asked to refresh. Occasionally participants read a word they had recently refreshed; these ‘match’ events were included to parallel *match* events in update trials. *Read* events corresponded to the non-match trials in update blocks. Participants did not make any button press responses in Refresh Blocks.

Across all scans there were 144 events of each type (update, match and read in the update task, and refresh, “match” and read in the refresh task).

#### fMRI data acquisition

All scans were collected on a Siemens 3T Trio scanner at the Magnetic Resonance Research Center, Yale University School of Medicine. The 6 fMRI experimental runs each lasted 9 minutes 18 seconds, for a total of 55 minutes, 48 seconds of functional data collection. Each block lasted 64.5 seconds with a temporally jittered interblock interval where a row of pluses remained on the screen for 3–7.5 seconds.

#### Update and refresh

Functional data were collected as T2*-weighted gradient echo, echo planar images (TR = 2000 ms, TE = 25 ms, Flip Angle = 80 degrees, voxel size 3.438×3.438 mm, 3 mm axial slice, 1 mm gap, FOV = 22 cm, matrix = 64×64) during the experimental task. A high-resolution T1-weighted anatomical scan was collected between the 3^rd^ and 4^th^ functional scan runs (TR = 2530 ms, TE = 3.34, Flip Angle = 7 degrees; FOV = 256×256).

#### Resting state scan session

Participants were told to keep their eyes open in the resting state (task free). In this scan session eight fMRI resting state runs lasted 6 minutes each.

All scans were also collected on a Siemens 3T Trio scanner at the Magnetic Resonance Research Center, Yale University School of Medicine. Resting state connectivity data were obtained using a gradient echo T2*-weighted gradient echo, echo planar images (TR = 1550 ms, TE = 30 ms, Flip Angle = 80 degrees, voxel size 3.4×3.4 mm, 6 mm axial slice, no gap, FOV = 22 cm, matrix = 64×64) during the experimental task. Data were motion corrected using SPM5 and slice time corrected. For the resting state scan session a high-resolution axial T1-weighted anatomical scan was collected after the functional scan runs (TR = 2530 ms, TE = 3.34, Flip Angle = 7 degrees; FOV = 256×256).

### Identification of seed regions

**Figure 2:**
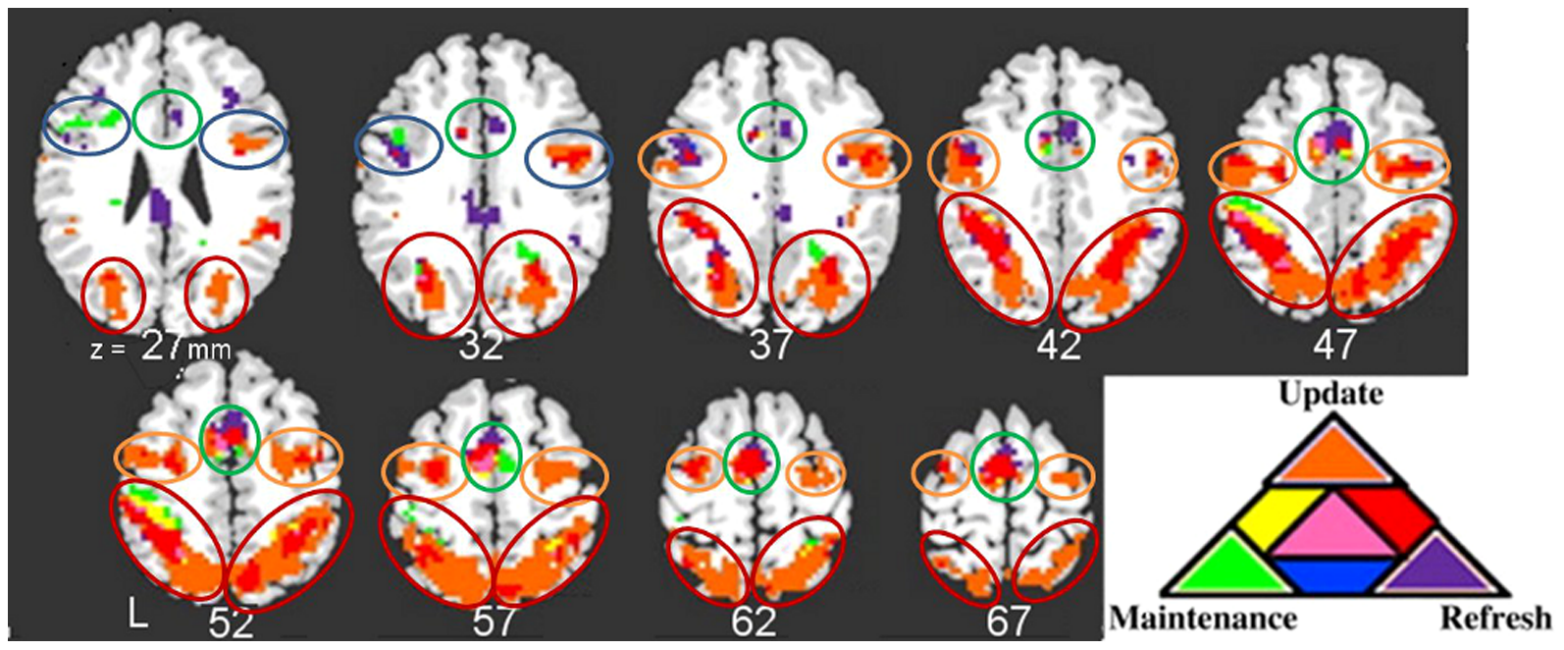
Seeds in the various major regions used for the functional connectivity analyses. Note: These seed regions were determined to be active to one or more of the update, maintenance and refresh component processes of working memory in a previous event-related experiment [1]. Within each major region, the differently colored areas were found to be differently responsive to one or a combination of events in the event-related analysis with update-responsive voxels colored as orange, refresh-responsive voxels colored as purple, voxels with sustained maintenance related activity shown in green, voxels showing responsiveness to two different events shown in yellow, red or blue, and voxels responsive to all event types shown in pink. Each differently active region was used as a seed in the functional connectivity analysis. The seeds were grouped into sets by the major regions of IFJ, SFJ, SMA and left and right parietal. Separate ANOVAs were run on data for each of these sets of seeds with each seed generating whole-brain voxel-wise functional connectivity values. IFJ seeds are circled in blue, SFJ in orange, SMA in green and parietal in red.

In the previous experiment [1] regions commonly active in many cognitive tasks (SMA, IFJ, SFJ and parietal) were shown to have subregions differentially active in component processes of working memory: update, refresh and maintenance. These subregions were used as seeds in the current functional connectivity analysis. To identify seed regions Roth et al (2009) conducted a series of t-tests to determine areas of the brain responsive to update, refresh, and maintenance They identified three individual voxelwise t-maps: Update versus control events, refresh versus control events, and update blocks versus refresh blocks. Maintenance was identified by the update block>refresh block contrast (since update but not refresh required sustained working memory maintenance; described in detail in Roth et al, 2009). Next, using AFNI software, an intersection map was generated by weighting equally the active voxels from each individual t-map and summing these to yield a map where different sums corresponded to different combinations of active conditions. The threshold for each of the three t-maps contributing to this overlap map was p<.01, providing a conservative method for determining areas of overlapping activation as each region of overlap had to pass the threshold for two or more t-tests. Seed regions were defined as areas of the brain active in one or more of the contrasts: update>control, refresh>control, or update block>refresh block. Individual participant data were transformed to standard, MNI space, to calculate group statistics. The resulting seeds provided functional subdivisions of major areas of cortex. These seeds are shown in Figure 2 and Table 1 (reproduced with permission from NeuroImage [1]). These regions are commonly active in many tasks and are often described as the ‘task positive’ regions.

**Table 1:** Regions used as seeds in the functional connectivity analysis.

			MNI Center of Mass (mm)		
Region	Volume (microlitres)	BA	X	Y	Z
**Update**					
**Prefrontal**					
L Precentral Gyrus/SFJ	4054	6	−50	−8	46
R Precentral Gyrus/SFJ	9000	6	38	−5	44
L SFJ	1487	6	−28	−8	56
**Medial**					
L SMA	216	6	−6	−18	67
R Superior SMA	432	6	8	−12	70
**Parietal**					
Bilateral Supremarginal Gyri, Intraparietal Sulcus, Primary and Secondary Visual Cortex	108867	40, 17, 18	6	−64	17
L SPL	216	40	−33	−38	40
**Refresh**					
**Prefrontal**					
L Precentral Sulcus	2027	6, 44	−49	2	31
R Precentral Gyrus	568	6	38	−4	39
L DLPFC	595	9, 46	−38	31	23
R DLPFC	649	9, 46	34	27	23
**Medial**					
Bilateral Posterior Cingulate	2757	23	−2	−31	29
Bilateral Anterior SMA	324	6	1	3	63
Bilateral SMA/ACC	3270	6, 32	2	12	44
**Parietal**					
L Intraparietal Sulcus	243	40	−29	−48	37
R Supramarginal Gyrus	216	40	50	−43	42
**Maintenance**					
**Prefrontal**					
L Postcentral Gyrus	703	2	−47	−27	50
L IFJ	514	9, 44	−45	12	28
L IFS	622	8	−30	15	22
**Medial**					
R SMA	595	6	7	−4	55
**Parietal**					
R Precuneus	703	7	22	−51	33
L Inferior Parietal/Postcentral Sulcus	324	40	−32	−38	52
**Overlap Update and Maintenance**					
**Parietal**					
L Inferior Parietal/Precentral Sulcus	1378	40	−41	−36	49
**Overlap Update and Refresh**					
**Temporal**					
R STG/MTG/Angular Gyrus	2784	21, 22, 40	52	−45	9
**Overlap of Update and Refresh**					
**Prefrontal**					
L Precentral Gyrus/IFJ	1216	6	−50	−2	42
R MFG/Precentral Gyrus/SFJ	1730	6	44	−3	39
L SFJ	1568	6	−32	−8	55
**Medial**					
Bilateral SMA	3757	6	−4	−4	62
**Parietal**					
L Inferior Parietal/Intraparietal Sulcus	7838	40	−38	−48	44
R Inferior Parietal/Intraparietal Sulcus	5514	40	33	−52	44
R Precuneus	1568	7	10	−70	52
**Overlap Update Refresh and Maintenance**					
**Medial**					
L SMA	1081	6	−6	−1	53
**Parietal**					
L SPL	405	7	−29	−57	44
L Parietal/Precentral Sulcus	378	40	−50	−33	46

These regions were active in update, refresh, maintenance, or active for any combination of these conditions. These excerpts from Table 1 and Supplementary Table 1 from Roth et al, 2009 [1], were reprinted with permission from NeuroImage, Elsevier.

Notes: R = Right; L = Left; ACC = Anterior Cingulate Cortex; IFJ = Inferior Frontal Junction (Junction of the Precentral Sulcus and Inferior Frontal Sulcus); IFS = Inferior Frontal Sulcus; IT = Inferior Temporal Gyrus; MFG = Middle Frontal Gyrus; SFJ = Superior Frontal Junction (Junction of the Precentral Sulcus and Superior Frontal Sulcus); SMA = Supplementary Motor Area; STG = Superior Temporal Gyrus. Brodmann areas (BA) are reported not only for the peak active voxel but for the entire active region. Only areas of overlap with volumes of of 200 microlitres or greater are included in this table. ** For the Refresh>Update contrast, there was a large cluster (85,191 microlitres) and coordinates for local maxima for sub-regions of this large cluster are reported instead of centers of mass.

In the present study these identified seeds were then grouped by the major region where they appeared (SMA, IFJ, SFJ and left and right parietal cortexes). The functional connectivity analyses described below were run separately for each of these sets of seeds regions grouped by major region.

### Functional connectivity analysis

Data were preprocessed differently from the task analysis to determine areas that were differentially active during task and rest. Data were slice time corrected and motion corrected with SPM5 (identical to data preprocessing for the seed identification analysis). Functional data were temporally high-pass filtered across volumes at 0.1 Hz in order to reduce the effects of physiologic noise such as respiration and heart rate, known to produce nuisance correlations in functional connectivity analysis. Anatomical and functional images were coregistered then morphed into MNI space using BioimageSuite software (http://www.bioimagesuite.org); identical to the data preprocessing for the seed identification analysis). A simultaneous regression analysis was conducted in order to examine interregional communication due to low frequency, state changes within the task, rather than high frequency changes due to the specific elements of the task. The robust event related response to each event within the task (update, refresh, read, match, button presses, control event cues, false alarm errors) was regressed from the data using a simultaneous regression with a standard hemodynamic response function (see [1]). The residual timecourse was used for the functional connectivity analysis. Functional connectivity analysis of the residual variance reflects the functional organization of the brain (i.e., ‘state’) during the period assessed. This working memory ‘state’ (i.e., where refresh and update were combined) was then compared to the resting state. The first 16 seconds of each experimental block were eliminated from the analysis in order to remove any effects of transitions between tasks.

Timecourses for each seed region were determined by averaging the voxel values, timepoint by timepoint, over all voxels within a seed region. The global mean was removed from all data. A 3^rd^ order polynomial function corrected for 2^nd^ and 3^rd^ order drift. A 3^rd^ order polynomial filter removes linear drift and 3^rd^ order drifts in the data. Data were subjected to a temporal smoothing function with a Gaussian kernel with a sigma of 1 timestep (that is, one TR). Separately for each block, a seed region's timecourse was used to calculate correlation coefficients with every other voxel's timecourse in the brain volume. R values were converted to Z-scores via Fischer's Z transform. Z-values were averaged across each block type. Individual participant data were smoothed with a three dimensional 4 mm FWHM Gaussian kernel.

For a separate group of 45 participants scanned during resting, data were concatenated across scan runs before connectivity was calculated [34]. Then resting state connectivity patterns for the same seed regions identified in the working memory tasks described above were calculated using similar methods as those described above.

#### Determining regions of differential functional connectivity between seeds and between task vs. rest

To find regions of the brain showing differential functional connectivity, the voxelwise Z-transformed correlation coefficients were entered into a series of ANOVAs to compare conditions (task versus rest, and seed regions (39 seeds)). The global mean signal was regressed from the data reducing the contribution of physiological noise to the functional connectivity results [35]. In order to examine fluctuations in activity not modulated by individual task demands, the connectivity values for update and refresh were averaged together to create a comparison of working memory tasks versus rest. These connectivity values for update and refresh were calculated from the residual variance after the effects of task were regressed from the data.

Seeds were grouped into sets (termed ‘major regions’ throughout the paper) of contiguously activated suprathreshold voxels, grouped by the major regions (SMA, IFJ, SFJ, left parietal and right parietal). Because there were a large number of seeds in parietal cortex, these seeds were grouped by left and right hemispheres for subsequent ANOVAs. Separate ANOVAs were calculated for each major region. These voxelwise ANOVAs identified regions showing a main effect of condition (task versus rest). These ANOVAs assessed a main effect reflecting regions of the brain where task and rest connectivity were different, a main effect of seed reflecting regions of the brain where connectivity differs across seeds, and a Condition (task versus rest) x Seed (11 seeds for SMA, 11 for left parietal, 8 for right parietal, 5 for IFJ, 4 for SFJ) interactions (p<0.01) identifying regions of the brain where connectivity varies both with seed and condition.

All functional connectivity analyses, including both positive and negative correlations, were corrected for multiple comparisons with the False Discovery Rate (FDR) method. For main effects of condition and the condition x seed interaction maps, an FDR corrected p value of .01 was used. For the maps showing the main effects of seed region there was extensive, strong functional connectivity necessitating an FDR corrected p value of 1×10^−19^.

#### Analyses of regions with differential functional connectivity

Planned comparison t-tests were conducted for all regions showing a significant main effect of seed, main effect of condition or condition x seed interaction. T-tests were computed to determine if functional connectivity values for task and rest were significantly greater than zero for each region of the brain connected to that seed, for each seed at the p<0.05 level.

### Open access

The data will be made available upon request via an open access institutional repository.

## Results

### Subregions within major activated regions engage different networks

**Figure 3:**
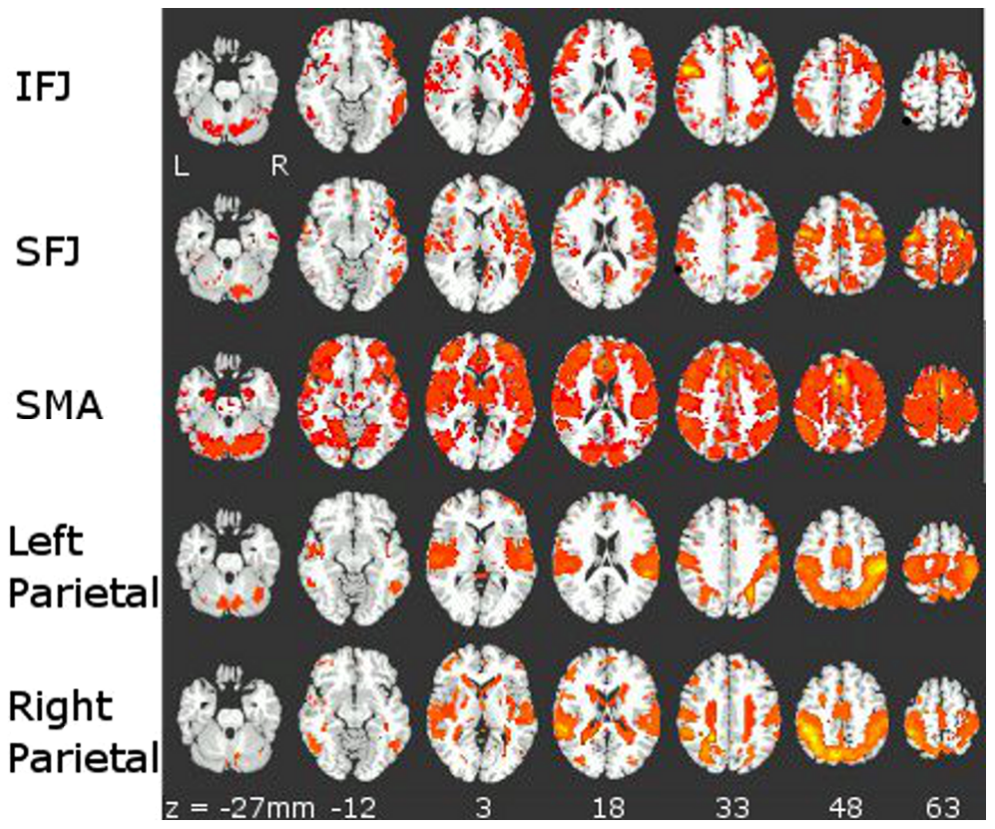
Maps for main effect of seed from the seed x task ANOVAs conducted separately on the z-transformed correlation coefficients from resting state and working memory task residual data for groups of seeds within 4 major regions: IFJ, SFJ, SMA and parietal cortex (p<1×10^−19^). Separate ANOVAs were conducted for each group of seeds for a total of five ANOVAs. Regions shown in shades of red to yellow have differential connectivity to different seeds within each of these four major regions. Functional connectivity to other brain regions (areas of significant connectivity to the seeds) changes with these small changes in the locations of the seeds, as measured across seeds in adjacent areas of cortex within a major region.

The sets of seeds were grouped by major regions of SMA, IFJ, SFJ, left and right parietal cortex to determine connectivity patterns to the entire brain. A series of condition (task versus rest) by seed ANOVAs were conducted separately for each set of seeds. These ANOVAs, using the correlation coefficients derived from the resting state dataset and from the residual variance from the working memory task dataset, showed a significant main effect of seed for each major region (see Figure 3), indicating differences in network connections.

**Figure 4:**
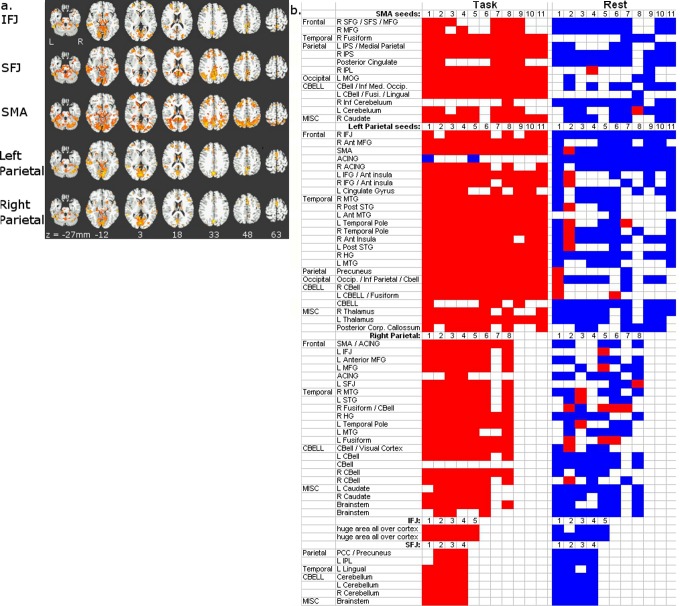
a. Maps for main effect of condition (task vs rest) from ANOVAs conducted separately on the z-transformed correlation coefficients from resting state and working memory task residual data for seeds within 5 major regions: IFJ, SFJ, SMA and left and right parietal cortex (p<.01). Regions in shades of red to yellow demonstrate significant differences in functional connectivity (connectivity between seeds and other brain regions) during rest versus during task. Therefore these regions are connected to these seeds differently during rest and task. b. Significant functional connections in task residual data and rest for example brain regions (20 voxels or greater in size) showing a main effect of condition for each set of seeds. This matrix is a summary of t-test results showing significant functional connectivity for task and rest, each versus a null distribution. Each cell represents the connectivity between a seed and another brain region. Results are grouped by the location of the major region containing the sets of seed: SMA, left parietal, right parietal, IFJ and SFJ. Results are further grouped by the location of the region (frontal, parietal, occipital, etc). The columns of the matrix represent the seeds within major region. Columns are further grouped by task or rest connectivity. An individual cell within the matrix denotes whether the connectivity between a given seed and another brain region had significantly functional connectivity for task or rest in a planned comparison t-test. Colored cells denote significant connectivity. Note that both in task and in rest there is significant connectivity for a majority of functional connections. Cells are color coded such that positive connectivity values are red, negative are blue. These results demonstrate a pattern of connectivity across different seeds and different regions such that there is usually positive connectivity between seeds and regions in task, and a heterogeneous pattern of connectivity in rest where some seeds showed positive connectivity to some regions, and negative connectivity to other regions. There is a heterogeneity of functional connectivity for a given seed where connectivity differs across regions connected to that seed. For example, looking at the functional connectivity between left parietal's seed 1 across regions, the colors change across the cells of the matrix indicating that this seed is not always connected to other regions in the same manner. Sometimes resting connectivity is positive, sometimes negative. Likewise, there is a similar heterogeneity of functional connectivity for a given region where connectivity differs across seeds. For example, the region the left temporal pole connected to seeds in left parietal has positive connectivity in rest to some seeds and negative connectivity for others.

Within each major region there was also a main effect of condition, indicating different network connections for task vs. rest (see Figure 4). All sets of seeds showed a main effect of condition in regions typically associated with greater activity in cognitive tasks than rest, and regions typically associated with less activity in task than rest [4], as well as task vs. rest differences in fusiform and parahippocampal gyri and visual cortex. When examining patterns of connectivity of connections between seeds and brain regions showing a main effect of condition, there was positive connectivity in the working memory tasks and negative connectivity during rest. In other words these seeds resulted in positive correlations with other areas during task and negative correlations during rest. The main findings are based on the main effect of seed and the interaction between condition and seed. Little emphasis is placed on the main effect of condition (a cross-session comparison of different participants).

At this threshold of p<.01, there was a significant seed by condition interaction for the seeds within the parietal cortex and SMA, but not for IFJ and SFJ. The lack of significant interactions between IFJ or SFJ seeds and other regions throughout the brain suggests that the networks connected to these seeds are engaged similarly during rest and task, or that any difference across task and rest is similar across seeds.

**Figure 5:**
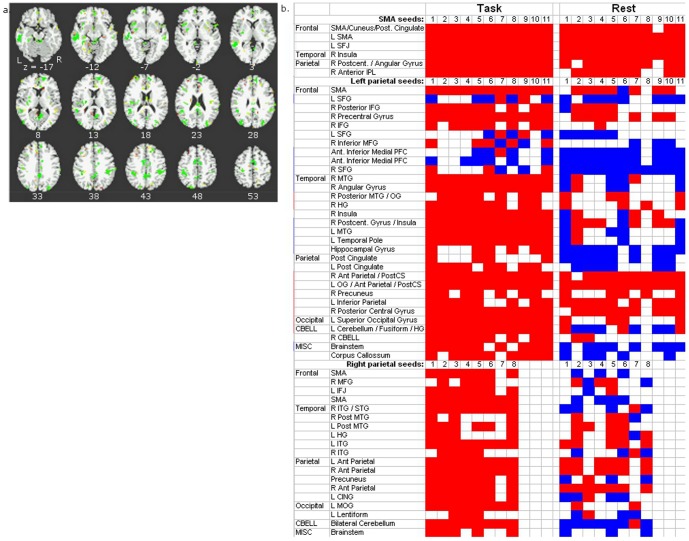
a. Map showing a condition (task vs rest) x seed interaction for one example set of seeds: the seeds in left parietal cortex (p<.01). Regions in green demonstrate significant interactions in functional connectivity between seeds and other brain regions across conditions and seeds. Therefore these regions are connected to these seeds differently during rest and task, differentially for different seeds. b. Significant functional connections in task residual data and rest for example regions (20 voxels or greater in size) showing task x seed interaction for each set of seeds. This matrix is a summary of t-test results showing significant functional connectivity for task and rest, each versus a null distribution. Each cell represents the connectivity between a seed and another brainregion. Results are grouped by the location of the major region containing the sets of seed: SMA, left and right parietal. Results are further grouped by the location of the brain region (frontal, parietal, occipital, etc). The columns of the matrix represent the seeds within major region. Columns are further grouped by task or rest connectivity. An individual cell within the matrix denotes whether the connectivity between a given seed and another brain region had significantly functional connectivity for task or rest in a planned comparison t-test. Colored cells denote significant connectivity. Note that both in task and in rest there is significant connectivity for a majority of functional connections. Cells are color coded such that positive connectivity values are red, negative are blue. There are different patterns of connectivity across different sets of seeds. For SMA there is positive connectivity between seeds and the other brain regions in task and rest. For right parietal there is positive connectivity between seeds and other brain regions in task and, in rest, there is a heterogeneous pattern of connectivity in rest where some seeds showed positive connectivity for some regions, and negative connectivity for other regions. Left parietal shows a more varied pattern of connectivity with a heterogeneous pattern of connectivity to several frontal regions both in task and rest. The connections between left parietal and regions throughout the rest of the brain have positive connectivity in task and heterogeneous connectivity in rest.

In both parietal cortex and SMA, the connectivity between particular seeds and particular other regions differed between the task residuals and rest (illustrated in Figure 5 for SMA and for left parietal cortex). This indicates that, for these seeds, there were different network configurations (i.e., patterns of functional connectivity) for task and rest conditions.

### Regions with significant task related connectivity also have significant connectivity during rest

Of the brain regions with significant connectivity in to seed regions during task (p<0.05; including only regions 20 voxels or greater in size), 58% of these regions showing a significant main effect of task also showed significant connectivity in rest (Figure 4). Of the regions showing significant task x seed interactions 64% of the regions with significant connectivity in task (p<0.05; 20 voxels or larger) also showed significant connectivity in rest (Figure 5). Connections that were significant both in task and in rest, and yet showed different connectivity patterns in task and rest, were presumed to engage the same network connections with functional connectivity modulated depending on state or task demands. Notably, the remaining connections (42% for those regions showing a main effect of task, and 36% for those regions showing a task x seed interaction) that were significant in task but not in rest suggest that the task engages functional connections that are not present in resting-state data at the thresholds used in this work.

### Connections between seeds and other brain regions may reflect network reconfiguration between rest and task

During task, correlations between seeds and brain regions that showed a main effect of condition (Figure 4a and 4b), were usually positive (left set of columns). In contrast, for rest, some seeds showed positive connectivity (right set of columns) with some regions (e.g., Fusiform and IFJ), and negative connectivity with other regions (e.g., MFG, SFG, IPL, Posterior Cingulate, cerebellum, STG). And, for a given brain region, connectivity was positive for some seeds and negative for others. Overall there are more consistently positive connections in task compared to rest.

For the brain regions showing a task by seed interaction (Figure 5a and 5b), there were different patterns of connectivity for different sets of seeds. For SMA seeds there was positive connectivity between seeds and regions throughout the brain in both task and rest, possibly reflecting engagement of these networks in both task and rest. Left parietal showed a varied pattern of connectivity to several frontal regions both in task and rest. Of the regions connected to left parietal seeds, one set of frontal regions showed negative connectivity in rest and heterogeneous connectivity in task. These connections enter a homogenous state of negative connectivity during rest; the networks are engaged during rest in an anticorrelated manner (i.e. have negative functional connectivity). The other frontal regions connected to left parietal show positive connectivity in both task and rest. Therefore these connections either are engaged in both task and rest, or do not change state across task and rest. The connections between left parietal and regions throughout the rest of the brain tend to have positive connectivity in task and heterogeneous connectivity in rest. For right parietal there was positive connectivity between seeds and other brain regions in task; in resting state some seeds showed positive connectivity to some regions, and negative connectivity to other regions, indicating a change of state to engage in the task.

## Discussion

Recent studies have found that adjacent functionally defined areas of cortex used as seed regions show different patterns of whole-brain functional connectivity [17], [16]. Our study defined adjacent regions from a study of component processes of working memory and then asked how the functional connectivity of seeds from these subregions compared to functional connectivity at rest. Different subregions of SMA, IFJ, SFJ and left and right parietal cortex (a set of regions known as the ‘task positive’ regions) were identified in a previous study as having event related responses to component processes of working memory [1]. The functional subregions were used here as seeds for a connectivity analysis comparing residual task data (i.e., with task evoked responses removed) with an independent sample of resting state data. In the present study, when these functionally defined adjacent regions were used as seeds, different networks were identified as being functionally connected to different seeds from within each of these regions (SMA, IFJ SFJ or parietal cortex). Because the event related response to each event type had been regressed from the data, lower frequency correlations in increases and decreases in activity (presumably, state related connectivity) remained. There was significant functional connectivity between these seeds and regions not activated by the task in the original event related analysis, suggesting that these functional connections were not driven by residual task-related variance in the regression analysis. The fact that different seeds have different functional connectivity to different regions demonstrates that directly adjacent functional subdivisions of SMA, IFJ, SFJ and parietal cortex, originally identified by event-related responses associated with component processes of working memory [1], are connected to different networks. The current results further support the hypothesis that these subregions represent functional divisions within larger areas found in many fMRI studies requiring executive functions.

Importantly, many of the functional connections identified in task performance were also significant in an independent sample of participants at rest (intrinsic). Notably, functional connectivity in task and rest was more similar for IFJ and SFJ regions than for SMA and parietal regions, suggesting greater modulation of intrinsic networks in task in SMA and parietal regions than in IFJ and SFJ. These findings are the first to demonstrate a division of commonly active regions based on component processes of executive function into regions that connect to separate and distinct networks, and that the patterns of network connectivity based on these seeds, changes between task and rest. These results have implications in understanding the roles of distinct subregions of cortex based on their participation in different functional networks.

### Many functional connections engaged in the task are intrinsic

There are correlations in activity between brain regions in the absence of an imposed task (i.e., resting state, or intrinsic, activity; for a review, see [36]). The cause of intrinsic correlations is not known. Intrinsic networks may reflect inherent structural connectivity and/or be the result of experience, as in a series of Hebbian synapses [37] formed over time, and/or reflect spontaneous cognitive activity [22], [23], [4]. Also, at rest, coactivation between areas even when the network is not engaged by a task may fortify connections and maintain networks important for common cognitive tasks. Comparing resting state connectivity with task connectivity is a way to identify these intrinsic functional networks in the brain.

A number of intrinsic networks have already been identified with major task divisions. For example, it has been demonstrated that functionally defined networks also have significant functional connectivity in rest for motor responses [18], vision [19], [20], audition [20], attention [23], [25], language [20], and long term memory [21], [24], [26]. Here we demonstrate that there are even more specific networks that can be identified by using seeds responsive to different component processes of executive function. That is, many of the cognitive networks connected to adjacent seeds in the current experiment showing significant task related connections were also significantly connected at rest, demonstrating that these task-driven networks are intrinsic.

It appears that functional connectivity across connections changes with changes in state – in the current experiment differences between being engaged in a working memory task and rest. When not engaged in the task, functional connectivity across connections appears to be heterogeneous (i.e., connections between the seeds of a major region and the brain regions connected to those seeds is a mixture of positive and negative connectivity). When that network is engaged in a task, the connectivity patterns become homogenous, where each connection between the seeds of a major region and the regions connected to those seeds all become positive or all become negative. For example, across seeds within a major region, the connectivity to other brain regions is positive for some conditions and negative for others in rest. During task these connections become positive (see figure 4B, connections between seeds located in right parietal and regions throughout frontal cortex). There are also regions whose connectivity is heterogeneous in task and homogeneous but negative in rest (see figure 5B connections between seeds located in left parietal cortex to regions throughout frontal cortex).

### Not all task networks are intrinsic

Some functional connections engaged in task were not significant at rest, suggesting that cognitive networks are not simply the engagement of intrinsic networks. For example, many connections shown in Figure 4 to have significant connectivity during task (left column, colored squares) do not have significant functional connectivity during rest (right column, uncolored squares). This pattern of connectivity for task and rest is consistent for many of the connections between seeds and other brain regions for all major regions (SMA, IFJ, SFJ, left parietal and right parietal). The significant connectivity found in the task residuals not seen at rest may represent a flexible assembly of networks that form for task-specific functions. This flexibility of network configuration may predict other cognitive abilities (for a review, see [38], or IQ, an open question proposed by Gray and Thompson [39]).

### Task positive networks connect positively to task negative as well as task positive regions during task

Our results show that subregions of traditional ‘task positive’ areas have positive correlations not only to the traditional ‘task positive’ but also to the ‘task negative’ (a.k.a ‘default mode’) regions during a working memory task. These network configurations shift from positive connectivity to heterogeneous connectivity (a mixture of positive and negative correlations) during rest (Figure 4). For example, as shown in Figure 4 in the column labeled ‘task’, the seeds in SMA, a typical task-positive region, are all positively correlated to the activity in right intraparietal sulcus, another task positive region. Also in the column labeled ‘task’ the activity in some of the same SMA seeds is also positively correlated with the activity in posterior cingulate, a traditionally task negative region. This pattern of positive correlations during the working memory task between seeds and traditionally task positive and task negative regions is seen throughout all sets of seeds. These results differ from typical functional connectivity results between task positive and task negative regions. Typically such analyses find negative connectivity between task positive seeds and task negative regions. The seemingly contradictory current results may result from the subdivision of task positive regions. Other studies have used larger portions of task positive regions as seeds. Here the task positive areas are subdivided into smaller units, responsive to components of a working memory task. These subregions within the traditional task positive region may connect positively to task negative regions. Similarly, the brain regions with significant connectivity to these seeds may also be more specific subregions of task negative regions. This speculation requires testing in future studies. However, consistent with our findings, some “task negative regions,” such as posterior cingulate, are sometimes seen as positively active in memory tasks in event related analyses [40], [41], [42], [43] It is not surprising then that subregions of areas such as posterior cingulate can be positively correlated to task positive regions in a functional connectivity analysis. These findings highlight that areas within the traditional task negative network, as well as regions in the traditional task positive network, participate in tasks under some circumstances.

In short, the parcellation of areas in IFJ, SFJ, SMA and parietal cortex into distinct functional subregions is supported by the current results. These subregions, when used as seeds in a functional connectivity analysis, show different functional connectivity networks. Further, task networks associated with seeds derived from an analysis of component processes of executive function can be seen in the resting state. For some of these, task demands appear to modulate activity in these intrinsic networks. Approximately half of the connections significant during task were significant during rest, indicating that some of the connections are intrinsic while others are recruited only in the service of the task. Some groups of connections became synchronized with positive functional connectivity between seeds and other brain regions during task, while these connections during rest were also significantly connected but showed heterogeneous patterns of connectivity. Some connections were correlated while others were anti-correlated, perhaps signifying a synchronization of network connections for functions more engaged in task or rest, respectively. Furthermore, during task, the low-frequency connections from the seeds to both task positive and task negative regions becomes positively correlated, suggesting that both the traditional task positive and task negative areas participate when a person is engaged in a cognitive task.
